# Are Functional Brain Networks Sensitive to High Phenylalanine in Adults With Phenylketonuria?

**DOI:** 10.1002/jmd2.70108

**Published:** 2026-07-03

**Authors:** Vanessa Vallesi, Yosuke Morishima, Laura Winiger, Roman Trepp, Regula Everts, Raphaela Muri

**Affiliations:** ^1^ Department of Diabetes, Endocrinology, Nutritional Medicine and Metabolism, Inselspital Bern University Hospital and University of Bern Bern Switzerland; ^2^ Translational Research Center, University Hospital of Psychiatry and Psychotherapy University of Bern Bern Switzerland; ^3^ Graduate School for Health Sciences University of Bern Bern Switzerland; ^4^ Translational Imaging Center (TIC) Swiss Institute for Translational and Entrepreneurial Medicine Bern Switzerland; ^5^ Division of Neuropaediatrics, Development and Rehabilitation, Department of Paediatrics, Inselspital Bern University Hospital and University of Bern Bern Switzerland

**Keywords:** dorsal attention network, intervention, phenylketonuria, randomized placebo‐controlled trial, resting‐state fMRI

## Abstract

In early‐treated adults with phenylketonuria (PKU), the effects of elevated phenylalanine (Phe) on functional brain networks remain poorly understood. While subacute structural brain changes have been reported, their functional significance remains unclear. Executive and attentional functions have been shown to be particularly sensitive to metabolic control in PKU. In this double‐blind, randomized, placebo‐controlled crossover trial, we investigated the effects of a 4‐week high‐Phe period on resting‐state functional connectivity using seed‐based analyses of the dorsal attention and frontoparietal networks, which are critically involved in executive and attentional functions. Twenty‐three adults with PKU (median age 35.3 years [IQR 12.0]; 43% female) were included. When baseline connectivity was considered, no significant differences in functional connectivity were observed between the Phe and placebo periods. Without baseline adjustment, exploratory analyses revealed increased functional connectivity of the right frontal eye field within the dorsal attention network with frontal regions. Changes in functional connectivity were neither associated with changes in metabolite concentrations nor in working memory, attention, or executive function. These findings suggest that subacute increases in Phe may induce subtle and potentially transient changes in functional brain networks, although their functional relevance and persistence over longer exposure periods remain unclear.

## Key Points

- After 4 weeks of high‐Phe exposure, findings in early‐treated adults with phenylketonuria could tentatively point toward alterations in attentional network connectivity.
- These functional connectivity alterations were not significant after accounting for baseline connectivity and were not associated with cognitive changes.

## 1 Introduction

Individuals with the rare genetic disorder phenylketonuria (PKU) are unable to convert phenylalanine (Phe) into tyrosine (Tyr) due to deficient or absent Phe hydroxylase activity. Newborns are typically screened within the first days of life, and if diagnosed, treatment is initiated immediately, consisting of a strict low‐Phe diet supplemented with Phe‐free amino acid mixtures. Elevated Phe during early brain development can lead to neurotoxic effects [1], promoting oxidative stress and disturbances in brain metabolism that contribute to neurological and cognitive impairments if untreated during childhood [2]. In early‐treated adults with PKU, returning to a strict diet has been associated with reversible changes in brain structure [3], suggesting that the effects of Phe exposure differ from those during early brain development. However, the mechanisms underlying these differences in the adult brain remain poorly understood, particularly regarding neural functioning and cognitive performance.

Executive function in adults with PKU has been reported to fall within the lower average range compared with normative data. Higher Phe concentrations during early childhood, as well as higher current Phe levels and greater Phe variability, were associated with poorer executive function performance [4]. Poorer dietary adherence in adults with PKU, reflected by higher median blood Phe levels over the previous year, has been associated with lower pallidum and brainstem volumes [5] and lower mean diffusivity in major white matter tracts, including the corpus callosum and the superior longitudinal fasciculus linking frontal, parietal, and temporal regions [6]. This pattern has been interpreted as potentially reflecting intramyelinic swelling or restricted water diffusion related to myelin abnormalities within white matter tracts involved in a wide range of cognitive functions. In a double‐blind, randomized, placebo‐controlled crossover trial conducted in the same participants as the present study, a 4‐week high‐Phe exposure led to reduced cortical thickness and increased white matter volume, with all changes returning to baseline after washout [7]. Consistently, axial, radial, and mean diffusivity decreased during high‐Phe exposure and normalized thereafter, indicating transient alterations in white matter microstructure [8]. While these findings suggest subacute plasticity in the adult PKU brain, it remains unclear whether such microstructural changes are accompanied by alterations in neural function.

Based on previous evidence linking metabolic control to executive functioning in PKU, we hypothesized that in patients with early‐treated PKU subacute increases in Phe levels during 4 weeks would be associated with alterations in functional connectivity within networks supporting executive and attentional processes, potentially accompanied by changes in cognitive performance.

## 2 Materials and Methods

This randomized, double‐blind, placebo‐controlled crossover trial compared the effects of high Phe (1500–3000 mg per day, depending on body weight and sex) and placebo on functional brain networks in early‐treated adults with classical PKU, as part of the Phenylalanine and Its Impact on Cognition (PICO) study [9]. The trial was conducted at the University Hospital of Bern, Switzerland, between July 2019 and June 2022. Patients were recruited in Switzerland, Germany, and Austria.

### 2.1 Ethics Approval

The study was approved by the local Ethics Committee of Bern (2018‐01609) and was conducted in accordance with the Declaration of Helsinki. The trial was preregistered on ClinicalTrials.gov (NCT03788343). All participants signed written informed consent.

### 2.2 Study Design

The study design included a measurement before and after each 4‐week intervention period, with a 4‐week washout period in between. Participants were randomly assigned to receive capsules containing either 1500–3000 mg Phe per day or pregelatinized starch as placebo in equivalent doses. This resulted in four visits. At each visit, concurrent venous blood Phe levels, questionnaires, cognitive assessments, and neuroimaging were obtained. All study team members and participants were blinded until completion of the initial analyses. The sample size was determined based on a priori power calculations (Supporting Information Section Data S1.1).

### 2.3 Eligibility Criteria

Inclusion criteria were a diagnosis of PKU identified by newborn screening, early initiation of dietary treatment, and age ≥ 18 years. Exclusion criteria included contraindications to magnetic resonance imaging (MRI), absence of a Phe‐restricted diet in the preceding 6 months or unwillingness to maintain it, Phe levels > 1600 μmol/L in the past 6 months, current pregnancy or lactation, inadequate contraception, use of medications affecting cognition, and relevant psychiatric or neurological comorbidities. Additional exclusion criteria have been reported previously [9].

### 2.4 Assessments

#### 2.4.1 Metabolic Assessments

Venous blood samples were obtained after an overnight fast before MRI acquisition. Plasma Phe, Tyr, and tryptophan (Trp) concentrations were measured using ion‐exchange liquid chromatography with postcolumn ninhydrin‐derivatization and photometric detection.

#### 2.4.2 Cognitive Assessments and Questionnaires

Following the MRI session, cognitive performance was assessed using a standardized neuropsychological test battery covering executive function, attention, general intellectual ability, and depressive symptoms (Supporting Information Section Data S1.2). See also Trepp et al. [10] for further information.

#### 2.4.3 MRI

MRI data were acquired on a 3‐T Siemens Prisma system using a 64‐channel head coil. Participants were scanned in a supine position and were instructed to remain still with their eyes closed during functional imaging, while staying awake. Acquisition parameters are detailed in Supporting Information Section Data S1.3.

### 2.5 Data Processing and Statistical Analysis

#### 2.5.1 Functional MRI Preprocessing and Analysis

Functional MRI (fMRI) analyses were conducted using CONN (version 22.a) [11, 12] with Statistical Parametric Mapping (SPM12) [13] within MATLAB (R2023b). Preprocessing steps included slice‐time correction, realignment to correct head motion, spatial normalization to the Montreal Neurological Institute (MNI) template, and smoothing with an isotropic 6‐mm full‐width‐at‐half‐maximum Gaussian kernel. Denoising regressed out physiological and motion‐related nuisance signals (CompCor [14] white matter and cerebrospinal fluid components, motion parameters and their first‐order derivatives, outlier volumes, and linear trends), and the residual blood‐oxygen‐level‐dependent (BOLD) time series were band‐pass filtered (0.008–0.09 Hz). Two participants were excluded for excessive motion. Detailed preprocessing and denoising specifications are provided in Supporting Information Section Data S1.4.

Seed‐based functional connectivity analyses were performed. Connectivity maps were computed for predefined networks using seed regions derived from the Human Connectome Project Independent Component Analysis (HCP‐ICA) atlas [11], including bilateral nodes of the dorsal attention network (frontal eye field [FEF] and intraparietal sulcus) and the frontoparietal network (lateral prefrontal and posterior parietal cortices). These networks were selected because they are critically involved in executive control and attentional processes, cognitive domains that have repeatedly been reported to differ in adults with early‐treated PKU [4, 15]. For each seed, the mean BOLD time series was extracted. Whole‐brain, voxel‐wise functional connectivity was then quantified by computing Pearson's correlation coefficients between this seed time series and the time series of every other gray matter voxel. These correlation coefficients were Fisher *z*‐transformed to improve normality for subsequent statistical analysis [16].

### 2.6 Statistical Analysis

Voxel‐wise seed‐based functional connectivity was assessed using general linear models (GLMs) [17] to compare placebo and Phe periods within participants. All models included age, sex, and condition order as covariates. Cluster‐level inference was performed under Gaussian random field theory [18, 19], using an initial cluster‐forming threshold of *p* < 0.001, followed by false discovery rate (FDR) correction at *q* < 0.05. Subsequent statistical analyses were conducted in R (version 4.3.3) [20] using RStudio (version 2023.12.1.402), primarily with the tidyverse [21] and stats [20] packages. For clusters surviving correction, we calculated 95% confidence intervals (CIs) and corresponding effect sizes using Cohen's *d* defined as the mean paired difference divided by the standard deviation of the differences. Effect sizes were interpreted using conventional thresholds (0.2 = small, 0.5 = medium, 0.8 = large) [22]. Exploratory analyses were conducted to examine associations between functional connectivity measures extracted from significant clusters and cognitive performance.

## 3 Results

### 3.1 Sample Characteristics

Twenty‐three adults with PKU with complete functional imaging data were included in the present analyses. Participants had a median age of 35.27 years (interquartile range [IQR]: 11.96), and 43.48% were female. Baseline demographic and clinical characteristics were comparable between randomization sequences (Phe–placebo vs. placebo–Phe), as reported previously [10]. Median baseline Phe concentrations were 787 μmol/L (IQR: 327) and increased to 1455 μmol/L (IQR: 298) during the Phe period, confirming effective metabolic manipulation.

### 3.2 Seed‐Based Functional Connectivity

When baseline connectivity was considered, no significant differences in functional connectivity were observed between the Phe and placebo periods for any of the predefined seed regions.

**FIGURE 1:**
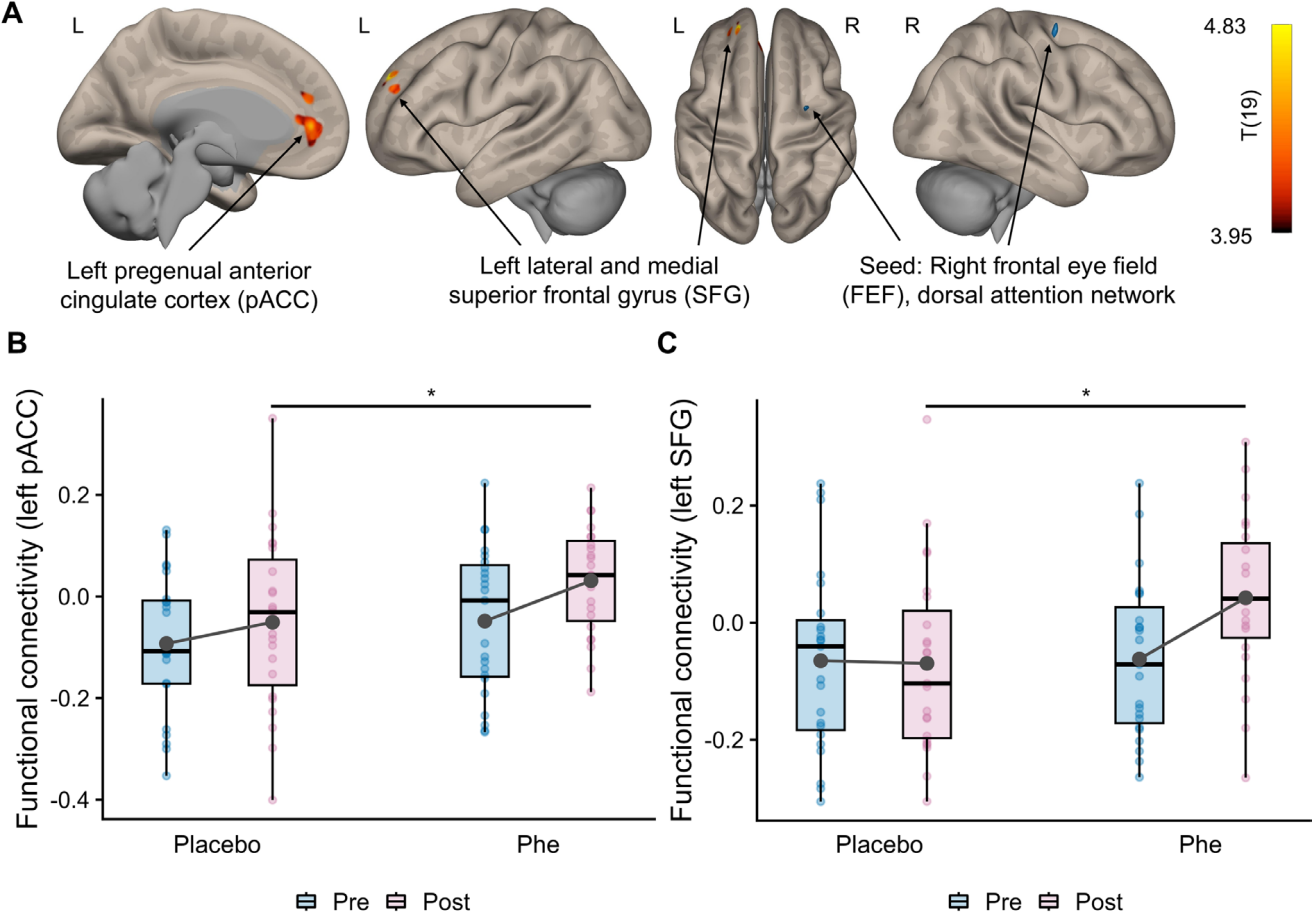
Seed‐based functional connectivity differences between the Phe and placebo periods. (A) Voxel‐wise seed‐based functional connectivity contrast comparing the Phe period with the placebo period in *n* = 23 participants with complete data. The right frontal eye field (FEF; dorsal attention network, shown in blue) was used as the seed region. Significant clusters in the left pregenual anterior cingulate cortex (pACC) and left superior frontal gyrus (SFG) are displayed, with color indicating *t*‐statistics. (B) Distributions of mean functional connectivity values extracted from the left pACC cluster for pre‐ (baseline) and postintervention measurements within the placebo and Phe periods. Individual participant values are shown as background points. Boxplots indicate the median and interquartile range, while filled circles and connecting lines denote the group mean functional connectivity from pre‐ to postintervention. (C) Corresponding pre and postintervention functional connectivity values extracted from the left SFG cluster, displayed using the same conventions as in (B). *p < 0.05 false discovery rate (FDR) corrected.

Without baseline adjustment, the direct postintervention comparison between the Phe and placebo periods revealed significantly higher functional connectivity for the right FEF of the dorsal attention network with a cluster in the left pregenual anterior cingulate cortex (pACC; *t*_(19)_ = 5.18, *p*_FDR_ = 0.028, 70 voxels, mean difference = 0.08, 95% CI [0.00, 0.16], Cohen's *d* = 0.45, peak MNI coordinate = −12, 42, 2) and a cluster spanning the left lateral and medial superior frontal gyrus (SFG; *t*_(19)_ = 5.61, *p*_FDR_ = 0.028, 76 voxels, mean difference = 0.11, 95% CI [0.04, 0.19], Cohen's *d* = 0.65, peak MNI coordinate = −20, 52, 28) (Figure 1). The left FEF and bilateral intraparietal sulcus of the dorsal attention network, as well as the bilateral prefrontal and posterior parietal cortices of the frontoparietal network, did not show significant differences in functional connectivity during the Phe period compared with the placebo period.

To assess the robustness of these findings, additional control analyses were performed. Functional connectivity did not differ between the two preintervention baseline periods (baseline Phe vs. baseline placebo), and no evidence for carry‐over effects was observed.

Changes in functional connectivity between conditions (Δfunctional connectivity; [Phe‐baseline] − [placebo‐baseline]) extracted from the FEF‐pACC and FEF‐SFG clusters were examined in relation to changes in cognitive performance (Δcognitive score; [Phe‐baseline] − [placebo‐baseline]) using linear regression models adjusted for age, sex, and treatment order. No significant associations were observed for working memory, sustained attention, or executive function (all *p* > 0.05; Table Data S1). Similarly, no significant associations were found between changes in functional connectivity and changes in metabolite levels (Phe, Trp, Tyr) (all *p* > 0.05).

## 4 Discussion

This randomized, double‐blind, crossover interventional study examining the effects of a 4‐week high Phe exposure provides limited evidence for altered functional connectivity within the dorsal attention network. Specifically, increased functional connectivity between the right FEF and frontal and medial prefrontal regions, including the pACC and SFG, was observed during the high Phe period. These effects were restricted to the right FEF and were not observed for other nodes of the dorsal attention or frontoparietal networks. Importantly, they were not associated with cognitive changes and were no longer significant when baseline connectivity prior to each intervention phase was considered. While these findings could tentatively point toward an early adaptive or compensatory neural response to increased Phe exposure, the lack of cognitive correlates and dependence on baseline adjustment warrant a cautious interpretation.

Our findings are broadly consistent with previous neuroimaging studies suggesting involvement of frontal brain regions in PKU. A task‐based fMRI study reported increased activation in frontal regions during inhibitory control [23]. A cross‐sectional perfusion study conducted in the same sample as the present study showed reduced blood flow in frontal territories [24], without evidence for an association with concurrent Phe levels. In contrast, a task‐based fMRI study using a working memory paradigm in this sample did identify an association between Phe levels and neural activity in frontal regions [25]. In contrast to these cross‐sectional approaches, the present study directly manipulated Phe levels. Despite this, only subtle functional connectivity effects were observed, suggesting that while frontal systems may be sensitive to PKU‐related alterations, the relationship between Phe levels and functional brain networks is not straightforward and may not be captured by subacute fluctuations in Phe.

Interventional studies in PKU have shown that neither a single high‐dose Phe administration during a cognitive control task [26] nor a 4‐week period of high Phe exposure during a working memory task including the present sample significantly altered task‐related BOLD activation [27]. Against this background, the observed resting‐state changes may reflect alterations in network organization not directly captured by task‐evoked activation. However, given the absence of cognitive effects and the sensitivity of the findings to baseline adjustment, it remains unclear whether these changes reflect meaningful functional adaptations or transient fluctuations.

Resting‐state functional organization in PKU remains relatively underexplored. In one of the few studies, functional connectivity alterations were observed within attention networks and the default mode network, but not in frontoparietal networks [28]. These findings were based on a higher criticism‐based approach capturing spatial activation patterns rather than functional connectivity. In the present study, changes were likewise observed within the dorsal attention network. Although these effects were subtle, the convergence with previous findings may point toward attention‐related networks as a potentially sensitive system in PKU. This could be of interest for future studies specifically targeting resting‐state network organization.

A recent hypothesis paper proposed that chronic hyperphenylalaninemia may disturb glycine and D‐serine homeostasis and thereby modulate NMDA receptor activity [29]. NMDA receptor‐mediated signaling plays a central role in synaptic plasticity and the coordination of distributed neural activity. As functional connectivity reflects the temporal coupling between brain regions, alterations in NMDA‐related neurotransmission could, in principle, influence large‐scale network organization. However, such mechanisms were not assessed in the present study and remain speculative.

The present findings reflect neural responses to a subacute (4‐week) increase in Phe levels. Functional brain alterations in PKU may evolve over longer timescales and not be fully captured by subacute manipulations. The sample size has limited statistical power to detect subtle connectivity effects and associations with cognition. Accordingly, the present results are regarded as hypothesis‐generating and in need of replication in larger, adequately powered cohorts. Furthermore, the median baseline Phe concentration of patients (787 μmol/L) exceeded the current European treatment target of 600 μmol/L; while the intervention produced a substantial further increase, a plateau effect at higher Phe levels cannot be fully excluded. Future studies targeting individuals with prolonged off‐diet exposure or longitudinal dietary changes may clarify whether network‐level alterations become more stable or behaviorally relevant over time.

## 5 Conclusion

After 4 weeks of high Phe exposure, the attentional network showed subtle changes in functional connectivity that were not associated with cognitive performance. These findings may reflect discrete or transient neural responses to elevated Phe levels. However, given the lack of robustness after baseline adjustment, it remains unclear whether prolonged exposure would result in more stable network changes and eventual behavioral consequences.

## Author Contributions

Conception and design: R.E., R.T., and R.M. Funding acquisition: R.E., R.T., and R.M. Data collection: R.M. Data analysis: V.V., Y.M., and R.M. Creating figures and tables: V.V. Drafting of the article: V.V. Reviewing and editing the article: Y.M., L.W., R.E., R.T., and R.M. Supervision: R.E., R.T., and R.M.

## Funding

The study was funded by the Swiss National Science Foundation (SNSF) with a project grant to R.E. (192706), and a SNF doc.CH grant awarded to R.M. (184453), the Vontobel Foundation (Switzerland), the Gottfried und Julia Bangerter‐Rhyner‐Stiftung (Switzerland), a Young Investigator Grant from the Inselspital Bern (CTU Grant) (Switzerland), the Nutricia Metabolics Research Fund (The Netherlands), and the Fondation Rolf Gaillard Pour la Recherche en Endocrinologie, Diabétologie et Métabolisme (Switzerland).

## Ethics Statement

All authors hereby state that they have complied with the principles of ethical publishing.

## Conflicts of Interest

The authors declare no conflicts of interest.

## Supporting information


**Data S1:** Supporting Information.
**Table S1:** Association between change in functional connectivity (ΔFC) and change in cognitive performance.

## Data Availability

The data that support the findings of this study are available on request from the corresponding author. The data are not publicly available due to privacy or ethical restrictions.
