# Supplementary material for: Are Functional Brain Networks Sensitive to High Phenylalanine in Adults With Phenylketonuria?
Source: JIMD Rep. 2026 Jul 3;67(4):e70108. doi: 10.1002/jmd2.70108 (PMC13330130; doi:10.1002/jmd2.70108)
Supplement: Supplementary file 1 — Data S1: Supporting Information. Table S1: Association between change in functional connectivity (ΔFC) and change in cognitive performance. [file JMD2-67-e70108-s001.docx]

**Supplementary Material**

**S1. Methods**

**S.1.1 Power Calculation**

The sample size was determined using a power calculation based on the primary endpoint of the PICO study, namely working memory performance^1^. The calculation targeted a non-inferiority margin of 4% using a paired design with 80% statistical power and a one-sided significance level of 5%, resulting in a required sample of 26 participants with PKU. To account for potential dropouts, the sample size was increased to 30 participants.

**S.1.2 Neuropsychological assessments**

Cognitive assessment included measures of: (1) executive functions including inhibition and cognitive flexibility, measured with the Color-Word Interference Test (Conditions 3 and 4) from the Delis-Kaplan Executive Function System (D-KEFS)^2^; (2) attention, measured with the Test of Attentional Performance (TAP), specifically indices of divided attention (omission errors) and sustained attention (response time variability)^3^; (3) working memory, assessed with an n-back task (accuracy rate) as conceptualized within the Miyake model of executive functions^4^; and (4) general intellectual ability at baseline, estimated from subtests of the Wechsler Adult Intelligence Scale-IV (WAIS-IV; Matrix Reasoning, Vocabulary, Arithmetic, Symbol Search)^5^. Depression symptom severity was quantified using the Beck Depression Inventory (BDI)^6^.

**S.1.3 MRI Protocol**

High-resolution T1-weighted images were acquired using an MPRAGE sequence (TE = 2.26 ms, TR = 1950 ms, inversion time = 900 ms, flip angle = 9°, slice thickness = 1 mm, in-plane resolution = 1 × 1 mm², field of view = 256 × 256 mm², 176 slices; acquisition time: 4:34 min).

Functional MRI data were collected using a multiband echo-planar imaging sequence (TE = 37 ms, TR = 1300 ms, flip angle = 52°, multiband factor = 4, voxel size = 2.2 mm isotropic, field of view = 230 × 230 mm², 300 volumes; scan duration: 6:39 min).

**S.1.4 Functional MRI Preprocessing and First-Level Analysis**

Image preprocessing was performed using standard procedures. Functional images were first realigned to the initial volume using a six-parameter rigid-body transformation and resampled with B-spline interpolation to correct for head motion and susceptibility-related distortions^7,8^. Slice timing correction was then applied using sinc interpolation, aligning all slices to the middle of the acquisition time^9,10^. Volumes exceeding a framewise displacement threshold of 0.9 mm were identified as outliers using the Artifact Detection Tools (ART) toolbox^11–13^.

Structural and functional images were segmented into grey matter, white matter, and cerebrospinal fluid and subsequently normalised to Montreal Neurological Institute (MNI) space with a 2 mm isotropic resolution, using SPM’s unified segmentation approach and the IXI-549 tissue probability template. Spatial smoothing of functional images was performed with a 6 mm full-width-at-half-maximum Gaussian kernel^11,14–16^.

Denoising of functional data included regression of nuisance signals, comprising principal components derived from white matter and cerebrospinal fluid signals (five components each, estimated using the CompCor method^17^), motion-related regressors (six realignment parameters and their first-order derivatives)^18^, identified outlier volumes, and linear trends. Temporal bandpass filtering was applied between 0.008 and 0.09 Hz^19^. Global signal regression was not performed in order to retain potentially meaningful neural variance contained within the global signal^20^.

Quality control procedures identified two participants with excessive motion or global signal fluctuations exceeding three standard deviations, who were excluded from further analyses. Following denoising, 95.5% of functional connectivity edges showed no significant association with motion, as assessed by permutation testing (p > 0.05), indicating effective removal of motion-related artifacts.

**S2. Results**

| Supplementary Table 1. Association between change in functional connectivity (ΔFC) and change in cognitive performance. | | | | |
| --- | --- | --- | --- | --- |
| Right frontal eye field (seed) → left pregenual anterior cingulate cortex | | | | |
| Outcome | N | ΔFC β (SE) | p-value | R^2^_adj_ |
| Δsustained attention (response time variability in ms) | 22 | -102.91 (69.60) | 0.158 | 0.19 |
| Δn-back (accuracy rate) | 23 | -2.90 (10.90) | 0.793 | -0.12 |
| Δcognitive flexibility (s) | 23 | -19.40 (29.31) | 0.516 | 0.08 |
| Right frontal eye field (seed) → left superior frontal gyrus | | | | |
| Outcome | N | ΔFC β (SE) | p-value | R^2^_adj_ |
| Δsustained attention (response time variability in ms) | 22 | 1.36 (73.09) | 0.985 | 0.09 |
| Δn-back (accuracy rate) | 23 | -16.62 (9.90) | 0.110 | 0.03 |
| Δcognitive flexibility (s) | 23 | -44.40 (26.95) | 0.117 | 0.18 |
| Note: The linear regression models tested the association between change in functional connectivity (ΔFC) and change in cognitive performance. Δ values represent the treatment effect calculated as (phenylalanine period - baseline) - (placebo period - baseline). All models were adjusted for age, sex, and treatment order. Abbreviations: FC, functional connectivity; SE, standard error. | | | | |

**S3. References**

1. Trepp R, Muri R, Abgottspon S, et al. Impact of phenylalanine on cognitive, cerebral, and neurometabolic parameters in adult patients with phenylketonuria (the PICO study): a randomized, placebo-controlled, crossover, noninferiority trial. *Trials*. 2020;21(1):178. doi:10.1186/s13063-019-4022-z

2. Delis D, Kaplan E, Kramer J. Delis-Kaplan Executive Function System (DKEFS). *Psychol Corp*. Published online 2001.

3. Zimmermann P, Fimm B. Testbatterie Zur Aufmerksamkeitsprüfung. *Psytest*. Published online 2009.

4. Miyake A, Friedman NP, Emerson MJ, Witzki AH, Howerter A, Wager TD. The Unity and Diversity of Executive Functions and Their Contributions to Complex “Frontal Lobe” Tasks: A Latent Variable Analysis. *Cognit Psychol*. 2000;41(1):49-100. doi:10.1006/cogp.1999.0734

5. Van Ool JS, Hurks PPM, Snoeijen-Schouwenaars FM, et al. Accuracy of WISC-III and WAIS-IV short forms in patients with neurological disorders. *Dev Neurorehabilitation*. 2018;21(2):101-107. doi:10.1080/17518423.2016.1277799

6. Beck AT, Ward CH, Mendelson M, Mock J, Erbauch J. Beck Depression Inventory (BDI). *APA PsycTests*. Published online 1961. doi:https://doi.org/10.1037/t00741-000

7. Andersson JLR, Hutton C, Ashburner J, Turner R, Friston K. Modeling Geometric Deformations in EPI Time Series. *NeuroImage*. 2001;13(5):903-919. doi:10.1006/nimg.2001.0746

8. Friston KarlJ, Ashburner J, Frith CD, Poline J ‐B., Heather JD, Frackowiak RSJ. Spatial registration and normalization of images. *Hum Brain Mapp*. 1995;3(3):165-189. doi:10.1002/hbm.460030303

9. Henson R, Büchel C, Josephs O, Friston K. The Slice-Timing Problem in Event-related fMRI. *NeuroImage*. 1999;9(125).

10. Sladky R, Friston KJ, Tröstl J, Cunnington R, Moser E, Windischberger C. Slice-timing effects and their correction in functional MRI. *NeuroImage*. 2011;58(2):588-594. doi:10.1016/j.neuroimage.2011.06.078

11. Nieto-Castanon A. Preparing fMRI Data for Statistical Analysis. *arXiv*. Preprint posted online 2022. doi:10.48550/ARXIV.2210.13564

12. Power JD, Mitra A, Laumann TO, Snyder AZ, Schlaggar BL, Petersen SE. Methods to detect, characterize, and remove motion artifact in resting state fMRI. *NeuroImage*. 2014;84:320-341. doi:10.1016/j.neuroimage.2013.08.048

13. Whitfield-Gabrieli S, Nieto-Castanon A, Ghosh S. Artifact detection tools (ART). Published online 2011.

14. Ashburner J. A fast diffeomorphic image registration algorithm. *NeuroImage*. 2007;38(1):95-113. doi:10.1016/j.neuroimage.2007.07.007

15. Ashburner J, Friston KJ. Unified segmentation. *NeuroImage*. 2005;26(3):839-851. doi:10.1016/j.neuroimage.2005.02.018

16. Calhoun VD, Wager TD, Krishnan A, et al. The impact of T1 versus EPI spatial normalization templates for fMRI data analyses. *Hum Brain Mapp*. 2017;38(11):5331-5342. doi:10.1002/hbm.23737

17. Behzadi Y, Restom K, Liau J, Liu TT. A component based noise correction method (CompCor) for BOLD and perfusion based fMRI. *NeuroImage*. 2007;37(1):90-101. doi:10.1016/j.neuroimage.2007.04.042

18. Friston KJ, Williams S, Howard R, Frackowiak RSJ, Turner R. Movement‐Related effects in fMRI time‐series. *Magn Reson Med*. 1996;35(3):346-355. doi:10.1002/mrm.1910350312

19. Hallquist MN, Hwang K, Luna B. The nuisance of nuisance regression: Spectral misspecification in a common approach to resting-state fMRI preprocessing reintroduces noise and obscures functional connectivity. *NeuroImage*. 2013;82:208-225. doi:10.1016/j.neuroimage.2013.05.116

20. Liu TT, Nalci A, Falahpour M. The global signal in fMRI: Nuisance or Information? *NeuroImage*. 2017;150:213-229. doi:10.1016/j.neuroimage.2017.02.036
